# Dysfunction of Small-Conductance Ca^2+^-Activated Potassium (SK) Channels Drives Amygdala Hyperexcitability and Neuropathic Pain Behaviors: Involvement of Epigenetic Mechanisms

**DOI:** 10.3390/cells13121055

**Published:** 2024-06-18

**Authors:** Vadim Yakhnitsa, Jeremy Thompson, Olga Ponomareva, Guangchen Ji, Takaki Kiritoshi, Lenin Mahimainathan, Deborah Molehin, Kevin Pruitt, Volker Neugebauer

**Affiliations:** 1 Department of Pharmacology and Neuroscience, Texas Tech University Health Sciences Center, Lubbock, TX 79430, USA; 2 Center of Excellence for Translational Neuroscience and Therapeutics, Texas Tech University Health Sciences Center, Lubbock, TX 79430, USA; 3 Department of Pathology, UT Southwestern Medical Center, Dallas, TX 75390, USA; 4 Department of Immunology and Molecular Microbiology, Texas Tech University Health Sciences Center, Lubbock, TX 79430, USA; 5 Department of Pharmacology, University of North Carolina at Chapel Hill, Chapel Hill, NC 27599, USA; 6 Garrison Institute on Aging, Texas Tech University Health Sciences Center, Lubbock, TX 79430, USA

**Keywords:** neuropathic pain, amygdala central nucleus, SK channels, hyperexcitability, neuroplasticity, epigenetic regulation

## Abstract

Neuroplasticity in the amygdala and its central nucleus (CeA) is linked to pain modulation and pain behaviors, but cellular mechanisms are not well understood. Here, we addressed the role of small-conductance Ca^2+^-activated potassium (SK) channels in pain-related amygdala plasticity. The facilitatory effects of the intra-CeA application of an SK channel blocker (apamin) on the pain behaviors of control rats were lost in a neuropathic pain model, whereas an SK channel activator (NS309) inhibited pain behaviors in neuropathic rats but not in sham controls, suggesting the loss of the inhibitory behavioral effects of amygdala SK channels. Brain slice electrophysiology found hyperexcitability of CeA neurons in the neuropathic pain condition due to the loss of SK channel-mediated medium afterhyperpolarization (mAHP), which was accompanied by decreased SK2 channel protein and mRNA expression, consistent with a pretranscriptional mechanisms. The underlying mechanisms involved the epigenetic silencing of the SK2 gene due to the increased DNA methylation of the CpG island of the SK2 promoter region and the change in methylated CpG sites in the CeA in neuropathic pain. This study identified the epigenetic dysregulation of SK channels in the amygdala (CeA) as a novel mechanism of neuropathic pain-related plasticity and behavior that could be targeted to control abnormally enhanced amygdala activity and chronic neuropathic pain.

## 1. Introduction

Neuroplasticity in the central nucleus of the amygdala (CeA) contributes to the emotional–affective aspect of pain and pain modulation [1,2]. The lateral and capsular divisions of the CeA (CeLC) receive nociceptive inputs from the parabrachial (PB) nucleus [3,4]. PB neuronal firing is increased in acute and subacute pain conditions [5,6,7], potentiating synaptic transmission and driving hyperexcitability in the right, but not left, CeA [8,9,10,11]. It remains elusive how hyperexcitability is sustained in the right CeA under chronic pain conditions. 

Small-conductance Ca^2+^-activated potassium (SK) channels regulate neuronal firing and responsiveness to synaptic input [12,13,14,15,16] through the afterhyperpolarization of medium duration (mAHP). Somatic SK channels are expressed throughout the nervous system, including in the CeA [17,18]. SK channels are also found on dendritic spines, where they can shunt excitatory synaptic potentials [19]. Blocking dendritic SK channels facilitates the induction of long-term potentiation in the hippocampus and lateral amygdala [20,21]. In the CeA, the expression of SK2 and SK3 subtypes is high, with a low expression of SK1 [17,18]. Apamin blocks all SK channels, with the SK2 subtype being the most sensitive [22,23]. 

CeA neurons include late-firing, regular-firing, and low-threshold bursting phenotypes [24,25,26,27,28,29,30]. The majority of low-threshold bursting cells become regular-firing with stronger depolarization [30], but a subset of bursting neurons show complete spike-frequency adaptation due to a prominent slow AHP [24,31]. Through the mAHP, SK channels regulate spike frequency in regular- and late-firing CeA neurons, which include PKCδ- and somatostatin-expressing neurons [27,28,29,32], but not in adaptive, also referred to as accommodating neurons [13,24]. CeLC neurons with similar firing properties are topographically organized based on their projection targets, and a major population of late-firing neurons projects to the periaqueductal gray [33]. 

SK channels are involved in learning [34,35], fear extinction memory [36], stress [37], neurodegeneration [35], epilepsy [38], peripheral and spinal nociception [39,40,41,42], and based on evidence from our work, also in pain processing in the CeLC. The blockade of SK channels in CeA decreased the antinociceptive effects of riluzole, a putative SK channel activator and neuroprotective drug [43], and riluzole-induced potentiation of mAHP in CeLC neurons in pain conditions [44]. SK protein levels in the CeA were decreased in a visceral pain model [45], in the basolateral amygdala in repeated stressor-induced pain [37], and in the hypothalamus in a neonatal model of visceral pain [46].

The role and cellular mechanisms of SK channel function in amygdala neuroplasticity in pain are not known. We hypothesized that in chronic pain, inhibitory SK channel function is compromised in the CeLC, resulting in hyperexcitability. The effects of pharmacological blockade or potentiation of SK channels in the amygdala on pain behaviors and the cellular properties of CeLC neurons indicated a loss of SK channel function in chronic neuropathic pain. SK channel dysfunction involved SK channel protein and mRNA downregulation, specifically in the CeA, as the result of pretranscriptional mechanisms, such as DNA methylation of the SK2 gene in the promoter region. 

## 2. Materials and Methods

### 2.1. Animals

Male Sprague Dawley rats purchased from Envigo, which weighed 120–150 g (4–6 weeks old) at the beginning of the experiment (see Section 2.2), were housed in a temperature-controlled room under a 12 h day/night cycle with food and water without restriction. At the time of testing, the rats weighed 200–300 g (8–10 weeks old). Only male rats were used in this study because the hypothesis was not about sex differences but about the role and regulation of SK channels in chronic pain, and the scientific premise was based on previous work from our group and others that was mostly performed using males [47]. The experimental procedures were approved by the Texas Tech University Health Sciences Center Institutional Animal Care and Use Committee (IACUC) and conformed to the guidelines of the National Institutes of Health (NIH) and the International Association for the Study of Pain (IASP).

### 2.2. Neuropathic Pain Model

A mononeuropathy of the left hind paw was induced using spinal nerve ligation (SNL), an established model of neuropathic pain [48]. The rats were anesthetized with isoflurane (3–5% induction, 2% maintenance). The left paraspinal muscles were dissected, and the L6 transverse vertebral process was removed. The underlying left L5 spinal nerve was identified and ligated using silk thread. The muscles were sutured, and the skin was clipped closed. Bacitracin antibiotic was applied during the wound healing process, and animals were monitored for several days after surgery for signs of distress. Control (sham) animals received the same surgical procedure and post-operative care, but not SNL. To ensure that chronic neuropathic pain was fully established, behavioral and electrophysiological experiments and tissue collection were conducted 4–5 weeks after surgery.

### 2.3. Behavior

The behavioral tests were performed in light- and temperature-controlled rooms with reduced levels of ambient noise and light.

#### 2.3.1. Mechanosensitivity

Withdrawal thresholds were assessed using von Frey monofilaments (Touch Test^®^, Stoelting Co., Wood Dale, IL, USA) corresponding to 0.4, 0.6, 1, 2, 4, 6, 8, and 15 g, as described previously [44]. The rats were placed in plastic chambers with a wire mesh floor. Testing started after 10 min of habituation with the 2 g filament pressed against the tarsal surface of the left hind paw. If a withdrawal response occurred, the next lowest filament was used; otherwise, the next highest monofilament was used. The 50% threshold was calculated using the up–down method [49,50]. 

#### 2.3.2. Vocalizations

To assess supraspinally organized emotional responses, audible (20 Hz–16 kHz) and ultrasonic (25 ± 4 kHz) vocalizations were measured. Here, 25 kHz was chosen because, in rodents, these calls reflect a negative emotional state [51,52]. Vocalizations were measured as described previously [53,54]. The rats were briefly anesthetized with isoflurane to allow for the gentle placement, in a slightly restrained position, in a custom-designed chamber (US Patent 7213538) with openings for the head and limbs. The animals were allowed to habituate to the recording chamber for at least 30 min. If any signs of discomfort were observed, the animals were removed, and testing resumed the following day. A condenser microphone and a bat detector were used to record audible and ultrasonic vocalizations. The signals were amplified and digitized (UltraVox; Noldus Information Technology Inc., Leesburg, VA, USA). Noxious vocalizations were evoked by compression of the left hind paw for 15 s, using calibrated forceps with a transducer displaying force in grams. Pressure was gradually increased until 600–1000 g/6 mm^2^, which is known to evoke nocifensive reflexes but reflects a near-threshold stimulus intensity [53,54]. Care was taken not to exceed 1000 g/6 mm^2^ to avoid injury to the paw. Vocalizations were recorded for 1 min beginning with the onset of the stimulation. Total vocalization durations were measured. Vocalizations were replicated twice during vehicle and drug applications to ensure the repeatability of the test and then averaged.

### 2.4. Electrophysiology

#### 2.4.1. Brain Slice Preparation

Brain slices containing the CeA were obtained as described previously [54]. Briefly, the brains were rapidly removed and immersed in sucrose-based oxygenated ice-cold cutting solution containing the following (in mM): 87 NaCl, 75 sucrose, 5 KCl, 21 MgCl_2_, 0.5 CaCl_2_, and 1.25 NaH_2_PO_4_, and 25 glucose. Coronal brain slices (400 µm) were prepared using a vibratome (Vibratome Series 1000 Plus (IMEB Inc., San Marcos, CA, USA) or Leica Biosystems VT1200 (Fisher Scientific, Waltham, MA, USA)) followed by incubation in oxygenated artificial CSF (aCSF) containing the following (in mM): 117 NaCl, 4.7 KCl, 1.2 NaH_2_PO_4_, 2.5 CaCl_2_, 1.2 MgCl_2_, 25 NaHCO_3_, and 11 glucose. After 1 h of incubation at room temperature, a single brain slice was transferred into a submerged-style recording chamber where it was superfused (2 mL/min, 32–35 °C) with oxygenated aCSF via a gravity-driven system. Two brain slices containing the CeA were used per animal. If drugs were applied, only one neuron was recorded in each slice, and a new slice was used for the next experimental protocol. 

#### 2.4.2. Patch-Clamp Recording of CeA Neurons in the Right Amygdala

Voltage and current clamp recordings in whole-cell configuration were obtained from visually identified cells in the right CeLC using DIC-IR microscopy (Olympus BX51), as described in detail previously [54]. Two internal solutions were used to fill borosilicate glass recording pipets (4–6 MΩ tip resistance). The potassium gluconate-based internal solution contained the following (in mM): 122 K-gluconate, 5 NaCl, 0.3 CaCl_2_, 2 MgCl_2_, 1 EGTA, 10 HEPES, 5 Na_2_-ATP, and 0.4 Na_3_-GTP. A methyl sulfate-based solution has been suggested to provide more stable Ca^2+^ homeostasis in the whole-cell configuration for afterhyperpolarization studies [55,56]. The potassium methyl sulfate-based internal solution contained the following (in mM): 135 KMeSO_4_, 8 NaCl, 10 HEPES, 2 Na_2_ATP, and 0.3 Na_3_GTP. pH was adjusted with KOH (7.2–7.3), and sucrose was used to correct osmolarity (280–290 mOsm/kg) of the internal solution. Electrical signals were processed with Axoclamp 2 (Axon Instruments, Foster City, CA, USA) or Multiclamp 700B (Molecular Devices, San Jose, CA, USA) amplifiers and Digidata 1440A or 1550B interfaces (Molecular Devices) using pClamp 10 (Molecular Devices) software. Series resistance was monitored, and if it changed by >20%, the neuron was discarded. Neuronal excitability was studied under a current clamp. Action potentials were evoked from −60 mV with 0.5 s depolarizing current steps of increasing amplitude (50 pA steps). The number of spikes at a given current step was used to evaluate neuronal excitability as a firing frequency–current (F–I) relationship.

The afterhyperpolarization of medium duration (mAHP) was evoked by a large depolarizing current step (1 s, 700 pA) in a current clamp from −60 mV. The mAHP amplitude was measured by averaging antipeak values in the mAHP portion of the tail voltage (within 100 ms of termination of current injection). The current underlying the mAHP (I_AHP_) was investigated using a voltage clamp by depolarizing the cell from a holding potential of −60 mV to +10 mV by a 1 s voltage step. I_AHP_ was measured as the peak outward tail current within 100 ms of voltage step termination. When afterdepolarization was observed, the mAHP amplitude was set to 0 mV. For afterhyperpolarization studies, both potassium gluconate and methyl sulfate-based internal solutions were used, and data were pooled for analysis because no difference was found in the effects of interventions on mAHP or I_AHP_ amplitudes. 

Evoked excitatory postsynaptic potentials were recorded using the current clamp mode at resting membrane potential. A bipolar tungsten stimulating electrode (FHC, Bowdoinham, ME, USA) was placed on the visually identified PB afferents-containing fiber tract dorsomedial to the CeA and lateral to the caudate–putamen area (see [57]). Square wave pulses (0.15 ms, 30–60% of amplitude required to evoke action potentials) were delivered via a stimulus isolation unit (A365, World Precision Instruments, Sarasota, FL, USA).

### 2.5. Drugs and Drug Application

Apamin was applied to inhibit SK channels. Apamin is considered a blocker of all SK channel subtypes and actually acts on the channel pore via an allosteric mechanism [22,58]. NS309, a highly potent and selective positive SK channel modulator (though not selective for individual SK channel subtypes) was used to activate SK channels. NS309 increases channel sensitivity to Ca^2+^ by binding to calmodulin, a calcium-sensing protein complexed with the SK channel [59,60,61]. Drugs were purchased from Tocris (RD Systems Inc., Minneapolis, MN, USA). All drug applications and neuronal recordings were carried out in the right CeA because of evidence for lateralization of pro-nociceptive signaling to the right CeA, with the left amygdala having limited, opposing, or no role in pain processing [8,62,63,64]. 

#### 2.5.1. Behavioral Experiments

Drug administration via stereotaxic microdialysis into the right CeA or offsite was used as described previously [65]. The striatum is an adjacent structure that does not project to CeA. The rats were anesthetized with isoflurane (2–4%), and a guide cannula was inserted according to the coordinates: 2 mm caudal to bregma, 4.3 mm right of midline, and 6.5 mm deep. For offsite controls, the striatum dorsal to the right CeA was targeted by implanting the guide cannula 5–5.5 mm deep. The guide cannula was fixed to the skull with dental acrylic (Plastic One, Roanoke, VA, USA). Bacitracin antibiotic was applied to the exposed tissue to prevent infection. The rats were allowed to recover from surgery for at least 24 h. Before the behavioral experiment, a microdialysis probe (CMA/Microdialysis, Solna, Sweden) extending 1 mm beyond the guide cannula was inserted and connected to an infusion pump (Harvard Apparatus, Holliston, MA, USA) using polyethylene tubing. The experiments began with the administration of aCSF (vehicle control) followed by microdialysis of the drug. Drug or aCSF was perfused for 15 min at a rate of 5 µL/min to establish tissue equilibrium before testing started, and the application continued during testing. The concentration of NS309 in the microdialysis fiber was 100-fold higher than the target concentration based on previous studies for similar compounds [66,67]. The effective concentration of apamin (1 µM in microdialysis fiber) for behavioral studies was established in our preliminary experiments. 

#### 2.5.2. Brain Slice Experiments

The concentrations used for NS309 (10 µM) and apamin (100 nM) are commonly used in brain slice experiments [13,16,68]. The drugs were applied via gravity-driven perfusion (~2 mL/min) for 15–20 min to establish equilibrium in the slice. aCSF served as vehicle control in all brain slice experiments. 

### 2.6. Histology

#### 2.6.1. Verification of Microdialysis Probe Locations

The rats were euthanized by decapitation (Harvard Apparatus Decapitator), and the brains were quickly removed and kept in paraformaldehyde at 4 °C overnight. The brains were transferred to 30% sucrose in 0.1 M phosphate-buffered saline (PBS) and kept at 4 °C for 48 h before they were frozen. The brains were sectioned (30 m) using a cryostat (Vibratome UltraPro 5000, American Instrument Exchange, Haverhill, MA, USA), mounted, subsequently stained with hematoxylin and eosin, and coverslipped. Microdialysis probe tip locations were identified from the stained sections and plotted on diagrams [69].

#### 2.6.2. Immunohistochemistry for SK2 Protein Detection in the Right CeLC

The rats were deeply anesthetized (4% isoflurane) and transcardially perfused with 0.1 M PBS, followed by 4% paraformaldehyde in PBS. The brains were removed and then kept at 4 °C overnight in 4% paraformaldehyde, cryoprotected in 30% sucrose for 48 h, embedded in OCT, and stored at −80 °C until use. Coronal sections (35 μm) containing the CeA at 1.8–2.6 mm posterior to Bregma were prepared on a cryostat (Leica VT1200S, Leica Biosystems, Leica Biosystems, Danvers, MA, USA). The sections were permeabilized in 0.3% Triton in PBS (PBST) for 10 min, blocked in 5% non-fat dry milk in PBS for 1 h, and incubated with the primary antibodies (α-SK2, Guinea Pig, Alomone AGP-045, 1:250 and α-NeuN, Mouse, Millipore MAB377, 1:500) in 5% BSA, 10% goat serum in PBS overnight. The sections were washed 3 × 5 min in PBST and incubated in secondary antibodies (Goat α-Guinea Pig Alexa488, A110730 and Chicken α-Mouse Alexa647, A21463, Thermo Fisher Scientific, Waltham, MA, USA) at room temperature for 2 h. After washing for 3 × 5 min in PBST, the sections were mounted in antifade media (Prolong. P36935, Thermo Fisher). All of the samples were stained at the same time and imaged with the same conditions with confocal microscopy (Olympus FV3000, Olympus Life Science, Waltham, MA, USA). 

### 2.7. Molecular Biology Experiments

#### 2.7.1. Tissue Preparation

The rats were euthanized by decapitation and the brains were removed quickly and placed in oxygenated ice cold aCSF. The right CeA and BLA tissues were identified and isolated using anatomical markers (optic chiasm, optic tract, intercalated cell mass, and external capsule) and collected from 500–1000 μm thick coronal brain slices (1.6–3.3 mm posterior to bregma) obtained using a Vibratome (Series 1000 Plus) in ice cold, oxygenated aCSF. The tissue was stored at −80 °C until use. 

#### 2.7.2. Western Blotting

CeA tissue samples from SNL and sham rats were prepared in a whole-cell lysate preparation. Proteins were separated using pre-cast 4–12% Bis-Tris gels (Invitrogen, Carlsbad, CA, USA), blotted onto nitrocellulose membranes, blocked with 5% non-fat milk in Tris-buffered saline with Tween 20 at room temperature for 2 h, and probed with primary antibodies (α-SK2 subunit, Guinea Pig, Alomone AGP-045, 1:1000 and α-GAPDH, Goat, R&D systems, AF5718, 1:2000) at 4 °C overnight and then in secondary polyclonal antibodies (Donkey α–Rabbit, IRDye 680LT, LI-COR Biosciences, 1:20,000 and Donkey α-Goat, IRDye 800, LI-COR Biosciences, 1:15,000) at room temperature for 3 h. Image Studio™ Lite software 4.0 (LI-COR Biosciences, Lincoln, NE, USA) was used for quantification. Blots were stripped and re-probed after pretreatment with the SK2 subunit control peptide as a negative control. 

#### 2.7.3. Reverse Transcription Polymerase Chain Reaction (RT-PCR)

SK2 mRNA levels were determined using RT-PCR, as described previously [70,71]. Briefly, total cellular RNA from tissue samples from sham or SNL rats was isolated using Trizol reagent (Invitrogen, Carlsbad, CA, USA) and reverse-transcribed to form cDNA using the iScript cDNA synthesis kit (Bio-Rad Laboratories, Hercules, CA, USA). Taqman gene expression assay (Applied Biosystems, Foster City, CA, USA) containing primer and probe sets specific for the rat SK2 subunit (Rn00570910_m1) and rat glyceraldehyde 3-phosphate dehydrogenase (GAPDH; Rn01775763_g1) were used. RT-PCR was performed in 386-well optical plates in a final volume of 10 µL containing 5 µL of TaqMan Universal Mastermix (Applied Biosystems), 20 pmol of respective primers and 1/10th of reverse-transcribed RNA. A CFX384 real-time system (Bio-Rad Laboratories, Hercules, CA, USA) with thermal cycling conditions consisting of 40 PCR cycles at 95 °C/15 s and 60 °C/1 min was used to determine the cycle threshold (C_t_) value. Samples containing the SK2 and the GAPDH primer set were run in duplicate, and the average of the two samples was used as the C_t_ value. The ΔΔC_t_ method was used for quantification. The GAPDH C_t_ was subtracted from the SK2 C_t_ value to calculate the ΔC_t_ value. The ΔΔC_t_ value was calculated by subtracting the average sham ΔC_t_ value from sham and SNL ΔC_t_ values, and the formula 2^−ΔΔC_t_^ was used to compare sham and SNL mRNA levels.

#### 2.7.4. Bisulfite Conversion of DNA and Methylation-Specific PCR

This study was conducted using 9 tissue samples from SNL and 9 tissue samples from sham rats. For each of these tissue samples, 10 clones were sequenced to identify the methylated sites using two sets of primers, i.e., 5 clones for each primer set. Genomic DNA was extracted from CeA tissue samples using the Quick DNA/RNA Miniprep kit (D7001, Zymo Research Corp., Irvine, CA, USA) by adhering to the manufacturer’s guidelines. The samples were homogenized using high-impact 1 mm Zirconium beads (D1032-10, Benchmark Scientific) in a BeadBug 6 Microtube Homogenizer (D1036, Benchmark Scientific, Sayreville, NJ, USA) at 4000 rpm for 45 s. Approximately 500 ng of DNA was bisulfite-treated using the EZ DNA Methylation kit (D5001, Zymo Research Corp.). Methylation-specific primers to the proximal regions of the SK2 promoter were generated using the MethPrimer version 2.0 bisulfite conversion primer design tool (http://www.urogene.org/methprimer2/index.html, accessed between January and May 2020), as shown in Table 1. The EpiScope MSP kit (R100A, Takara Bio, San Jose, CA, USA) was used to amplify ~40 ng bisulfite-converted DNA using PCR initial denaturation conditions of 95 °C/30 s, 98 °C/5 s, annealing conditions of 60 °C/38 cycles and extension at 72 °C/1 min. Amplicons were analyzed on 3% agarose gel and visualized using a Gel Doc EZ gel documentation system (1708270, Bio-Rad).

#### 2.7.5. Bisulfite Sequencing Specific PCR and Cloning

Bisulfite-specific primers to the proximal SK2 promoter regions were obtained from MethPrimer version 2.0 (Beijing, China) as shown in Table 1. The EpiScope MSP kit (R100A, Takara Bio) was used to amplify ~40 ng of bisulfite-converted DNA using PCR initial denaturation conditions of 95 °C/30 s, 98 °C/5 s, annealing conditions of 60 °C/40 cycles and extension at 72 °C/1 min. The amplicons were analyzed on 2% agarose gel and visualized using a Benchtop Single UV transilluminator (95-0447-01, UVP), and the bands were excised. PCR products were gel-purified using the QIAquick gel extraction kit (28704, Qiagen, Germantown, MD, USA) and cloned into the pCRII vector using the Dual Promoter TA cloning kit (45-0007LT, Invitrogen) following the manufacturer’s protocol. The ligation reaction was purified with QIthe Aquick PCR Purification kit (28104, Qiagen) prior to transformation into INVαF’ chemically competent cells (C202003, Invitrogen, Waltham, MA, USA). *E. coli* cells were sApread on ampicillin agar plates (Q60120, Invitrogen) and treated with 80 µg/mL X-gal solution (X1220, Teknova) for the blue–white selection of colonies. Agar plates were incubated at 37 °C overnight for bacterial growth. White colonies were picked after 16–18 h of incubation into Luria Broth (LB) ampicillin media (Q60020, Invitrogen), and bacteria were allowed to grow overnight in a shaking incubator (New Brunswick Scientific, Aurora, CO, USA) at 225 rpm at 37 °C. The plasmids were purified using the QIAprep Spin Miniprep kit (27104, Qiagen). 

#### 2.7.6. Bisulfite DNA Sequencing and Analysis

The plasmids were digested at 37 °C/1 h with BamH1 (R31316S) and Xho1 (R0146S) restriction enzymes (New England Biolabs, Ipswich, MA, USA) and analyzed via 1% agarose gel electrophoresis to confirm the presence of inserts. Positive plasmid samples were sent to Genewiz for Sanger sequencing using M13 reverse primers, and bisulfite sequences were analyzed for methylation marks using the Quantification tool for methylation analysis (QUMA, http://quma.cdb.riken.jp/ (accessed June-December 2020). The following formula was used to calculate the percentage: methylation number of positive clones at the CpG site/total number of clones sequenced for SNL or sham × 100.

Predictions of transcription factors binding sites on SK2 promoter regions were made using the Alibaba2 gene regulation program, which constructs matrices from TRANSFAC 4.0 sites (http://gene-regulation.com/pub/programs/alibaba2/, accessed in June 2020).

### 2.8. Statistical Analysis and Scientific Rigor

GraphPad Prism software 10.2.3 (Graph-Pad Software, San Diego, CA, USA) was used for data analysis and presentation. The data are presented as the mean ± SEM. Two-tailed Student’s *t*-test (paired or unpaired) was used to compare two sets of normally distributed data tested using Shapiro–Wilk and Kolmogorov–Smirnov tests and with equal variances. For multiple comparisons, ordinary two-way ANOVA with Bonferroni post-tests was used. Two-way repeated measures ANOVA with Fisher’s uncorrelated post-tests was selected as the most appropriate for the before–after drug studies. Fisher’s LSD does not correct for multiple comparisons but is the only option with a mixed ANOVA design. For multiple comparisons, Fisher’s LSD is preferable to *t*-tests because it pools standard deviations from all groups. Effect sizes were estimated with G*Power software 3.1 (Dusseldorf, Germany) [72] using pilot data to obtain statistical significance at an alpha level of 0.05 for a power of 80%. Appropriate statistical analysis is indicated in the text and figure legends. Statistical significance was accepted at the level of *p* < 0.05. 

For scientific rigor, rats of equal age were randomly assigned to sham and SNL groups. The experimenters were blinded to the treatments of rats for SK2 expression and DNA methylation analysis. It was not possible to be blinded to SNL/sham surgeries in behavioral and electrophysiological experiments and, therefore, several experimenters independently conducted behavioral tests and electrophysiological recordings, and the data were pooled for quantitative analysis to avoid biases.

## 3. Results

### 3.1. Loss of SK Channel-Mediated Control of Pain Behaviors and Neuronal Excitability in Neuropathic Pain

**Figure 1:**
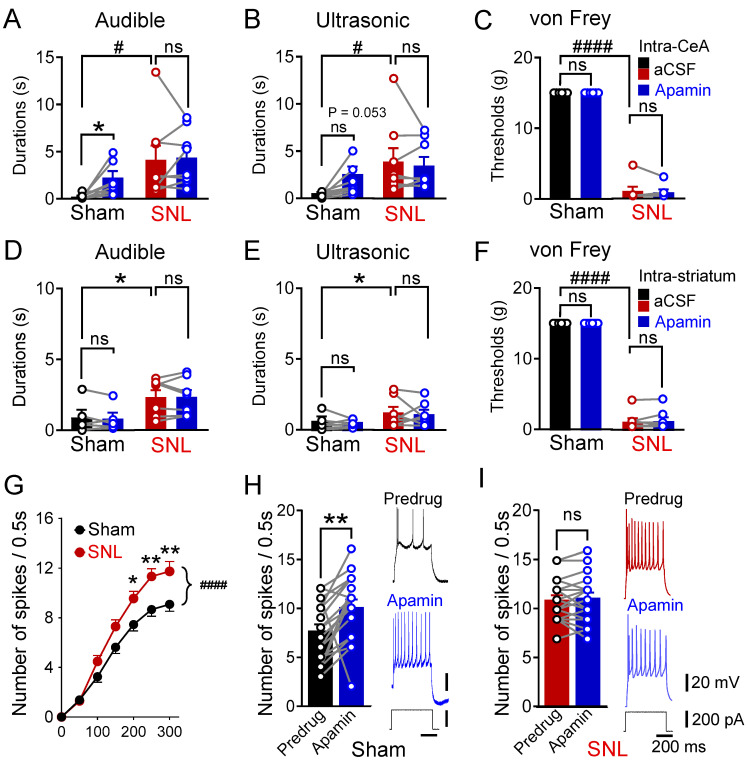
Blockade of amygdalar SK channels provokes neuropathic pain-like behaviors in sham rats by increasing the excitability of regular-firing neurons in the CeLC. (**A**,**B**). Durations of audible (**A**) and ultrasonic (**B**) vocalizations evoked via noxious mechanical stimulation of the left hind paw with calibrated forceps increased after intra-amygdalar SK channel blockade (1 µM apamin in microdialysis probe) in sham (audible: * *p* < 0.05; ultrasonic: ns, not significant, *p* = 0.053, *n* = eight rats) but not neuropathic rats 4 weeks after left spinal nerve ligation, SNL (ns, *p* > 0.05, *n* = eight rats, two-way repeated measures ANOVA with uncorrected Fisher’s LSD test). Note that audible and ultrasonic vocalizations significantly increased after SNL (audible: # *p* < 0.05 F _(1,14)_ = 6.73; ultrasonic: # *p* < 0.05, F _(1,14)_ = 5.386, main effect of SNL, *n* = eight rats per group, two-way ANOVA). (**C**) Mechanosensitivity in the von Frey test was not affected by intra-amygdalar microdialysis of an SK channel blocker (apamin 1 µM) in sham and in SNL rats (ns, *p* > 0.05, *n* = six sham rats, *n* = seven SNL rats, two-way ANOVA repeated measures with uncorrected Fisher’s LSD test). Note that mechanosensitivity significantly increased after SNL surgery #### *p* < 0.0001, F _(1,11)_ = 688.7, main effect of SNL, *n* = six sham rats, *n* = seven SNL rats, two-way ANOVA). D, E, F. SK channel blockade (1 µM apamin) in the striatum as a placement control had no effect on the durations of audible (**D**), ultrasonic (**E**) vocalizations evoked via noxious mechanical stimulation of the left hind paw with calibrated forceps, and mechanosensitivity (**F**) measured with von Frey filaments in sham and SNL rats (ns *p* > 0.05; *n* = six sham rats, *n* = seven SNL rats, two-way repeated measures ANOVA with uncorrected Fisher’s LSD test). Note that vocalizations and mechanosensitivity significantly increased after SNL (audible: * *p* < 0.05, F _(1,11)_ = 6.451; ultrasonic: *, *p* < 0.05, F _(1,14)_ = 5.5.832; mechanosensitivity: #### *p* < 0.0001, F _(1,11)_ = 603.8, main effect of SNL, *n* = six sham rats, *n* = seven SNL rats, two-way ANOVA). (**G**). Neuronal excitability measured using the F-I relationship evoked by depolarizing current injections of increasing magnitude (100 pA) was significantly increased (####, *p* < 0.0001, F_1,343_ = 30.5, main effect of SNL, *, ** *p* < 0.05–0.01, Bonferroni post-tests, two-way ANOVA) in slices from the SNL rats (*n* = 27 neurons) as compared to the sham controls (*n* = 24 neurons). (**H**). Apamin (100 nM) significantly increased firing frequency evoked by a 200 pA depolarizing current injection in CeLC neurons in slices from sham rats (** *p* < 0.01, paired *t*-test, *n* = 20 neurons). (**I**) In slices from SNL rats, the application of apamin (100 nM) did not further increase firing frequency in CeLC neurons (ns, *p* > 0.05, paired *t*-test, *n* = 20 neurons).

In agreement with previous studies [73,74], left spinal nerve ligation (SNL) generated pain-like behaviors that were studied here at the 4-week time point after surgery. The duration of vocalizations evoked by near-threshold noxious compression of the left hind paw with calibrated forceps and mechanical sensitivity tested with von Frey filaments applied to the plantar surface of the left hind paw (see Section 2.3) increased significantly in neuropathic rats compared to the sham control rats (Figure 1A–C, audible: # *p* < 0.05, F _(1,14)_ = 6.73; ultrasonic: *p* < 0.05, F _(1,14)_ = 5.386, main effect of SNL, *n* = 8 rats per group; von Frey: *p* < 0.0001, F _(1,11)_ = 688.7, main effect of SNL, *n* = 6 sham rats, *n* = 7 SNL rats; two-way ANOVA). 

The goal of this study was to understand the function of SK channels in the CeLC in chronic neuropathic pain. To do so, we first determined and compared the effect of a specific SK channel blocker (apamin) delivered stereotaxically via microdialysis into the right CeLC under control and neuropathic pain conditions. The right CeA was targeted because of evidence for lateralization of pain signaling to the right CeA [8]. In the sham control rats, apamin (15 min, 1 µM in the microdialysis probe; tissue concentration is 100-fold lower, see Section 2.5) significantly increased near-threshold noxious stimulation-evoked audible vocalizations and led to a non-significant increase in ultrasonic vocalizations (Figure 1A,B, audible: *p* < 0.05; ultrasonic: *p* = 0.053, *n* = eight rats per group, two-way repeated measures ANOVA with uncorrected Fisher’s LSD post-tests) without affecting mechanosensitivity (Figure 1C, von Frey: *p* > 0.05, *n* = six rats, two-way repeated measures ANOVA with uncorrected Fisher’s LSD post-tests). In SNL rats, intra-CeA administration of apamin had no effect on the increased vocalizations and mechanosensitivity (Figure 1A−C, *p* > 0.05, vocalizations: *n* = eight rats; von Frey: *p* > 0.05, *n* = seven rats, two-way repeated measures ANOVA with uncorrected Fisher’s LSD post-tests). Importantly, off-site microdialysis of apamin into the striatum had no significant effects on vocalizations and mechanosensitivity in either sham or SNL rats (Figure 1D−F, ns *p* > 0.05, *n* = six sham rats, *n* = seven SNL rats, two-way repeated measures ANOVA with uncorrected Fisher’s LSD post-test). The data suggest that under normal conditions, SK channels in the CeA are intrinsically active to attenuate emotional responses to acute noxious stimuli, but this function is lost in the chronic neuropathic pain state.

To determine neuronal mechanisms, we recorded a total of 75 neurons in the right CeLC using patch clamp electrophysiology in brain slices obtained from sham and SNL rats 4 weeks post surgery. All of the neurons were identified as receiving presumed PB input evoked by electrical synaptic stimulation (see Section 2.4). Action potentials were evoked via depolarizing current injections (0.5 s), and neurons were classified as regular spiking or adapting types. Regular-spiking neurons discharged repeatedly during depolarization, while adaptive cells showed high-frequency firing at the start of depolarization and developed spike frequency adaptation [24,30]. Excitability was measured as the number of spikes evoked by current injections. The resulting frequency–current (F–I) functions revealed increased excitability of regular-firing neurons in brain slices from SNL rats (*n* = 27 neurons) as compared to the sham rats (*n* = 24 neurons). This difference was significant (Figure 1G, *p* < 0.0001, F_1,343_ = 30.5, main effect of pain, two-way ANOVA). The blockade of SK channels with bath application of apamin (100 nM) increased the excitability of regular-firing neurons in brain slices from sham rats, where apamin increased the number of action potentials evoked by a 200 pA depolarizing current injection to 130% of the pre-drug values (Figure 1H, *p* < 0.01, paired *t*-test, *n* = 19 neurons). In contrast, apamin had no effect on the increased excitability of regular spiking neurons in brain slices obtained from neuropathic rats (Figure 1I, *p* > 0.05, paired *t*-test, *n* = 20 neurons). Individual examples show the apamin-induced increase in discharge frequency in the sham control condition (Figure 1H) but no additional effect of apamin on the number of action potentials evoked in a brain slice from an SNL rat (Figure 1I).

### 3.2. Loss of SK Channel Function (mAHP) in Regular-Firing Amygdala Neurons in Neuropathic Pain

**Figure 2:**
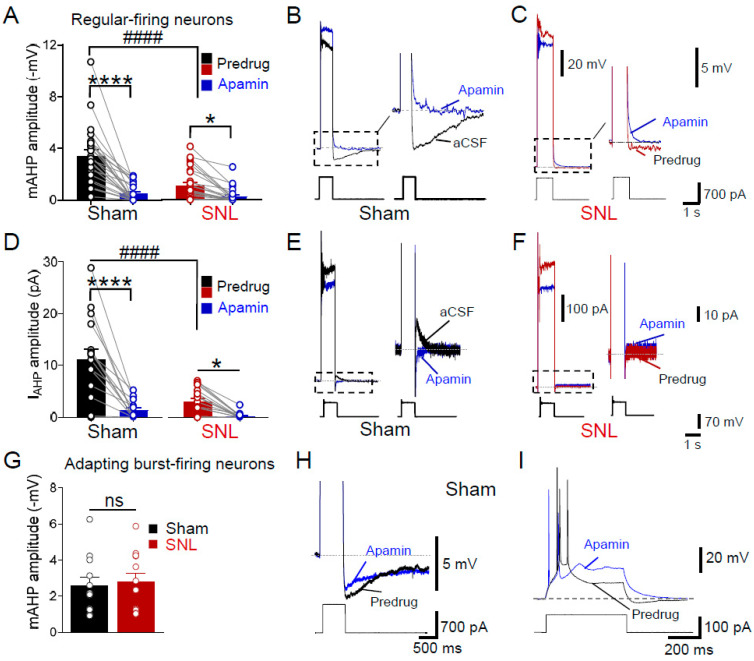
**Loss of SK channel function (mAHP) in regular-firing neurons in the CeLC in chronic neuropathic pain.** (**A**) Medium after-hyperpolarization (mAHP) evoked via current injection (700 pA, 1 s) was blocked in regular-spiking CeLC neurons in brain slices from neuropathic rats (4 weeks after spinal nerve ligation, SNL) as compared to sham controls (#### *p* < 0.0001, F _(1,49)_ = 18.69, main effect of SNL, two-way ANOVA, sham rats: *n* = 24 neurons; SNL rats: *n* = 27 neurons). Apamin (100 nM) blocked mAHP in slices from sham rats (**** *p* < 0.0001, *n* = 24 neurons) and reduced mAHP in slices from SNL rats (*n* = 27 neurons, * *p* < 0.05, two-way repeated measures ANOVA with uncorrected Fisher’s LSD post-test). (**B**,**C**) Traces show prominent mAHP in a brain slice from a sham rat (**B**) and no mAHP in a slice from an SNL rat (**C**). Dashed boxes depict magnified traces on the right. Voltage traces were truncated at higher magnification. (**D**). Current underlying mAHP (I_AHP_) evoked by 1 s depolarizing voltage steps to +10 mV was significantly decreased in CeLC regular-spiking neurons in brain slices from SNL rats compared to sham controls (#### *p* < 0.0001, F _(1,31)_ = 15.25, main effect of SNL, sham rats: *n* = 16 neurons; SNL rats: *n* = 17 neurons; two-way ANOVA). Apamin (100 nM) blocked I_AHP_ in brain slices from sham rats (**** *p* < 0.0001, *n* = 16 neurons) and decreased mAHP in SNL rats (* *p* < 0.05, *n* = 17 neurons, two-way repeated measures ANOVA with uncorrected Fisher’s LSD test). (**E**,**F**) Traces show I_AHP_ in CeLC neurons in brain slices from sham (**E**) and SNL rats (**F**). (**G**) Amplitude of mAHP evoked in adapting burst-firing cells via current injection (700 pA, 1 s) was not different between sham and SNL groups (ns, *p* > 0.05, *n* = 12 neurons per group, unpaired *t*-test). (**H**) Traces show long-lasting AHP in a low threshold bursting cell. Apamin (100 nM) blocked mAHP but not a slow AHP in these neurons. (**I**) Representative traces of apamin-insensitive spiking evoked by depolarizing current injection (500 ms, 100 pA) in a low threshold bursting cell.

The outflow of K^+^ through SK channels contributes to the generation of an afterhyperpolarization of medium duration (mAHP) that, under normal conditions, follows a train of action potentials to regulate neuronal firing frequency. In our experiments, SK channel-mediated mAHPs were evoked with a 700 pA depolarizing current step for 1 s from a membrane potential of −60 mV. The mAHP amplitude in regular-firing CeLC neurons in brain slices obtained from SNL rats was ~3.2-fold smaller than that of neurons from the sham controls (Figure 2A, *p* < 0.0001, F _(1,49)_ = 18.69; two-way ANOVA, sham rats: *n* = 24 neurons, SNL rats: *n* = 27 neurons). The blockade of SK channels with apamin (100 nM) significantly decreased mAHP in regular-spiking neurons in slices obtained from sham rats (Figure 2A, *p* < 0.0001, *n* = 24 neurons) and decreased any remaining mAHP in neurons from SNL rats (Figure 2A, *p* < 0.05, *n* = 27 neurons, two-way repeated measures ANOVA with uncorrected Fisher’s LSD test). Voltage responses of individual regular-firing neurons to a 1 s depolarizing step demonstrated prominent apamin-sensitive mAHP in slices obtained from a sham rat (Figure 2B) but not in the SNL model (Figure 2C). Thus, the blockade of mAHP under control conditions mimicked the near loss of mAHP in the neuropathic state.

The potassium current underlying mAHP (**I_AHP_**) was evoked at −60 mV using a voltage clamp with a 70 mV depolarizing step for 1 s. The **I_AHP_** amplitude in regular-spiking CeLC neurons recorded in brain slices from SNL rats was decreased compared to that in neurons from the sham control rats (3.13 ± 0.64 pA in SNL vs. 11.1 ± 2.04 pA in sham controls). This difference was significant (Figure 2D, *p* < 0.0001, F _(1,31)_ = 15.25, main effect of SNL, two-way ANOVA, sham rats: *n* = 16 neurons, SNL rats: *n* = 17 neurons). Apamin (100 nM) strongly decreased I_AHP_ in neurons in slices obtained from sham rats (Figure 2D, *p* < 0.0001, *n* = 16 neurons) as well as any remaining I_AHP_ in neurons from SNL rats (Figure 2D, *p* < 0.05, *n* = 17 neurons, two-way repeated measures ANOVA with Fisher’s uncorrected LSD post-tests). Current traces show the apamin-sensitive I_AHP_ in a regular-spiking CeLC neuron from a sham rat (Figure 2E) and loss of the I_AHP_ in a neuron from an SNL rat (Figure 2F).

Adapting burst-firing neurons, a common cell type in the CeA [24,31], were also studied here in the CeLC, both in brain slices from sham and SNL rats (*n* = 12 neurons per group). A depolarizing current step in these neurons evoked not only an mAHP but also a large, slow AHP decaying over several seconds. Interestingly, the mAHP amplitude in these neurons was not different between sham and SNL groups (*p* > 0.05, unpaired *t*-test; Figure 2G). Individual examples of an adapting burst-firing neuron show that apamin (100 nM) blocked the mAHP component but not the slow AHP (Figure 2H) and did not affect the firing properties (Figure 2I).

The data suggest a cell-type-specific loss of SK channel function in the CeA (CeLC) in a neuropathic pain model, which is evident from the impaired mAHP/I_AHP_ and lack of responsiveness to apamin, resulting in increased neuronal excitability in regular-firing CeLC neurons. In contrast, the mAHP present in adapting cells remained unchanged in the pain state and had no impact on their firing properties as tested with apamin, arguing against the contribution of this cell type to pain-related signaling and neuroplasticity in the CeA. Since no SK channel dysfunction was observed in bursting neurons in the neuropathic pain condition, we focused on regular-spiking neurons for subsequent studies using rescue strategies.

### 3.3. Selective SK Channel Activation Inhibits Neuropathic Pain Behaviors Due to Partial Rescue of mAHP

**Figure 3:**
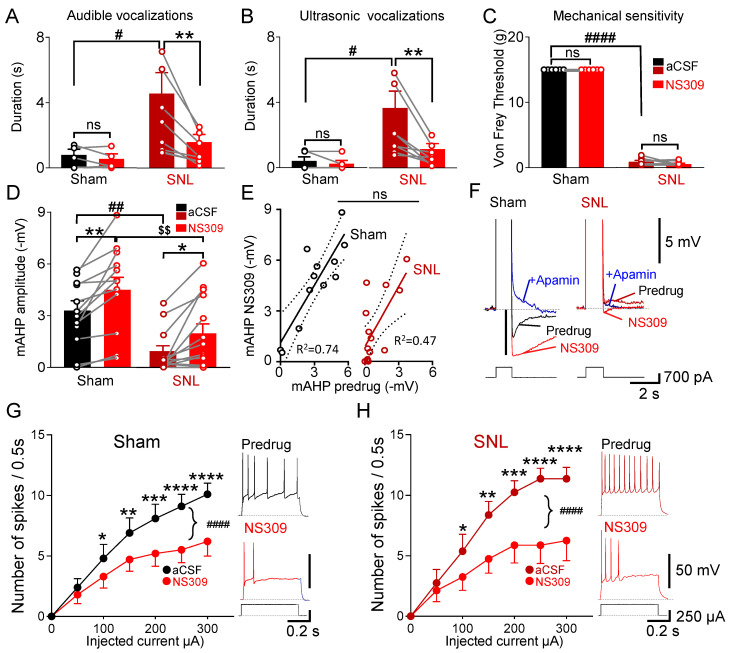
Selective activation of amygdalar SK channels inhibits affective behaviors and reduces excitability by restoring mAHP in CeLC in chronic neuropathic pain. (**A**–**C**). Audible (**A**) and ultrasonic (**B**) vocalizations evoked via the noxious compression of the left hind paw were significantly reduced via intra-CeA administration of a selective SK channel activator (NS309, 1 mM in microdialysis probe) in neuropathic (SNL) but not in sham rats (sham: ns *p* > 0.05; *n* = 5 rats; SNL: ** *p* < 0.01, *n* = 8 rats, two-way repeated measures ANOVA with uncorrected Fisher’s LSD test). Note, significantly increased pre-NS309 vocalizations in SNL rats compared to sham controls (audible: # *p* < 0.05, F _(1,11)_ = 5.02; ultrasonic: # *p* < 0.05, F _(1,11)_ = 5.80; main effect of SNL, two-way ANOVA, *n* = 5 sham rats, *n* = 8 SNL rats). Withdrawal thresholds (**C**) tested with von Frey monofilaments were not changed via the intra-amygdalar application of NS309 in sham (*n*–6 rats) and in SNL rats (*n* = 7 rats, ns, *p* > 0.05, two-way repeated measures ANOVA with uncorrected Fisher’s LSD test). Note the significantly decreased pre-NS309 withdrawal thresholds in SNL rats compared to sham controls (#### *p* < 0.0001, F _(1,11)_ = 11268, main effect of SNL, two-way ANOVA, *n* = 6 sham rats, *n* = 7 SNL rats). (**D**). In brain slice experiments, superfusion of NS309 (10 µM) increased mAHP in CeLC neurons in brain slices from sham (** *p* < 0.01, *n* = 12 neurons) and SNL rats (* *p* < 0.05, *n* = 14 neurons, two-way repeated measures ANOVA with uncorrected Fisher’s LSD test). Note the significantly smaller pre-NS309 mAHP amplitude in brain slices from SNL rats as compared to sham controls (## *p* < 0.01, F _(1,24)_ = 10.73, main effect of SNL, *n* = 12 sham rats, *n* = 14 SNL neurons, two-way ANOVA); mAHPs in the SNL model remained significantly smaller after activation with NS309 ($$ *p* < 0.01, sham: *n* = 12 neurons, SNL: *n* = 14 neurons, two-way repeated measures ANOVA with uncorrected Fisher’s LSD test). (**E**). Amplitudes of mAHP before and after activation with NS309 remained positively correlated in sham (R^2^ = 0.74) and SNL (R^2^ = 0.47) rats. Slopes were not significantly different (ns, *p* > 0.05, 2-tailed linear regression; *n* = 12 sham neurons, *n* = 14 SNL neurons). (**F**). mAHP traces before and after the application of NS309 (10 µM) followed by apamin (100 nM). (**G**,**H**). NS309 (10 µM) significantly decreased neuronal excitability measured as F–I relationship evoked by depolarizing current injections (50 pA steps) in slices from sham rats (####, *p* < 0.0001, F_1,63_ = 58.1, main effect of NS309, *, **, ***, **** *p* < 0.05–0.0001, Bonferroni post-tests, two-way mixed ANOVA, *n* = 10 neurons). (**G**) and SNL rats (####, *p* < 0.0001, F_1,49_ = 60.3, main effect of NS309, *, **, ***, **** *p* < 0.05–0.0001, Bonferroni post-tests, two-way ANOVA, *n* = 8 neuron). (**H**). Note that excitability was increased in slices from SNL rats as compared sham controls (*p* < 0.05, F_1,49_ = 4.9, main effect of SNL, mixed design ANOVA, *n* = 10 neurons from sham, *n* = 8 neurons from SNL rats). Activation of SK channels with NS309 decreased excitability to similar values in both groups (*p* > 0.05, F_1,112_ = 0.12, main effect of SNL, mixed design ANOVA, sham: *n* = 10 neurons, SNL: *n* = 8 neurons).

We assessed pain behaviors after the intra-CeA administration of a potent and selective SK channel activator (NS309) that acts by increasing the calcium sensitivity of the channel [60,75]. The stereotaxic administration of NS309 (1 mM, 15 min) into the right CeA did not affect audible and ultrasonic vocalizations evoked via the noxious compression of the left hind paw in sham controls (Figure 3A,B, *p* > 0.05; *n* = five rats) but significantly inhibited vocalizations in SNL rats (Figure 3A,B, *p* < 0.01, two-way repeated measures ANOVA with uncorrected Fisher’s LSD post-test; *n* = eight rats). Intra-amygdalar NS309 had no effect on the von Frey withdrawal thresholds in either group (*n* = six sham; *n* = seven SNL rats) compared to the pre-drug values (Figure 3C, *p* > 0.05, two-way repeated measures ANOVA with uncorrected Fisher’s LSD post-test), indicating the inhibition of averse-affective pain responses but not mechanosensitivity.

In order to study the mechanisms underlying the behavioral effects of selective SK channel activation, whole-cell patch clamp recordings were made of regular-firing neurons in the CeLC. mAHP amplitude significantly decreased in neurons recorded in brain slices obtained from SNL rats (*n* = 14 neurons) as compared to the sham controls (*n* = 12 neurons) (Figure 3D, *p* < 0.01, F _(1,24)_ = 10.73, main effect of SNL, *n* = 12 sham rats, *n* = 14 SNL neurons, two-way ANOVA). Bath application of NS309 (10 µM) significantly increased the mAHP amplitude in neurons in brain slices from sham rats (Figure 3D, *p* < 0.01, *n* = 12 neurons) and from SNL rats (Figure 3D, *p* < 0.05, *n* = 14 neurons, two-way repeated measures ANOVA with Fisher’s uncorrected LSD post-test) as compared to the pre-drug values. After activation with NS309, the mAHP recorded in brain slices obtained from the sham rats was significantly larger than in the SNL condition (Figure 3D, *p* < 0.01, sham: *n* = 12 neurons, SNL: *n* = 14 neurons, two-way repeated measures ANOVA with uncorrected Fisher’s LSD post-test), perhaps suggesting a change in the availability of functional SK channels in the neuropathic pain condition.

Accordingly, correlation analysis found that the larger the pre-NS309 mAHP was, the stronger the potentiation by the SK channel activator. There was a linear relationship between the mAHP amplitude and the effect of NS309. Although slopes of the regression lines were not statistically different (*p* > 0.05, F _(1,22)_ = 0.01, linear regression analysis), the correlation was stronger in neurons recorded in brain slices from sham rats (*n* = 12 neurons) compared to the SNL rats (*n* = 14 neurons, Figure 3E, sham, R^2^ = 0.74; SNL, R^2^ = 0.47). Where tested, the effect of NS309 was blocked by the subsequent application of an SK channel blocker (apamin, 100 nM), as demonstrated by the individual traces in Figure 3F.

The activation of SK channels with the bath application of NS309 (10µM) decreased the excitability of regular-firing neurons in brain slices from sham rats (Figure 3G, *p* < 0.0001, F_1,63_ = 58.1, main effect of drug, *n* = 10 neurons) and in slices from SNL rats (Figure 3H, *p* < 0.0001, F_1,49_ = 60.3, main effect of NS309, *, **, ***, **** *p* < 0.05–0.0001, Bonferroni post-tests, two-way mixed ANOVA, *n* = 8 neurons).

**Figure 4:**
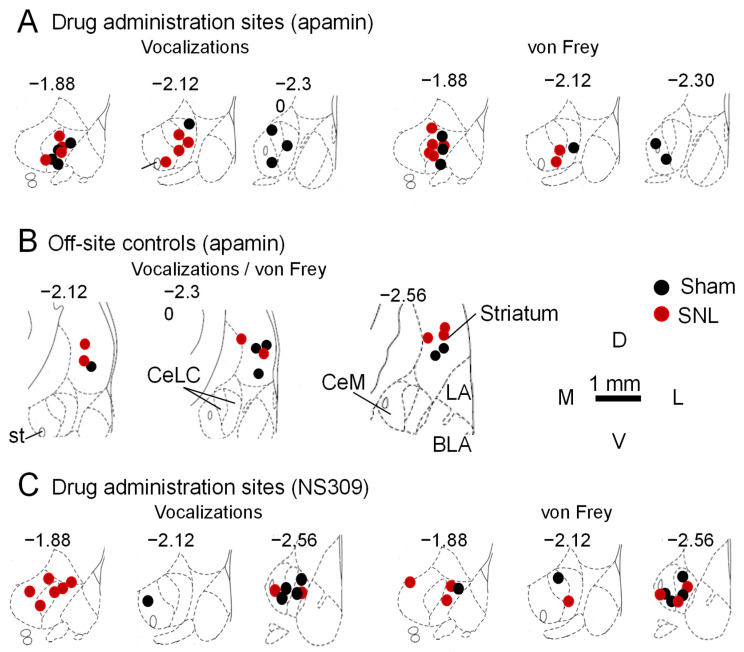
Locations of microdialysis probe tips for stereotaxic drug administration. (**A**–**C**). To match the recording sites in electrophysiological experiments, the lateral-capsular amygdala (CeLC) was targeted for microdialysis in behavioral experiments. Tip locations were assessed after testing the effects of SK channel blocker apamin or activator NS309 on vocalizations induced via the noxious compression of the left hind paw and mechanosensitivity tested with von Frey filaments. Symbols show the positions of the microdialysis probe tips in the CeA (**A**,**C**) and in the striatum as a placement control (**B**). Diagrams show coronal sections of the right rat amygdala (adapted from [69]). Numbers indicate distance (in mm) from caudal to bregma. Abbreviations: BLA, basolateral amygdala; CeLC, lateral-capsular CeA; CeM, central medial amygdala; LA, lateral amygdala; st, stria terminalis.

Positions of microdialysis probe tips for drug application into CeA or striatum in behavioral experiments were verified histologically and plotted on diagrams adapted from a rat brain atlas [69] (Figure 4).

### 3.4. Decreased SK2 Subunit Expression in Neuropathic Pain State Is Localized to CeA

**Figure 5:**
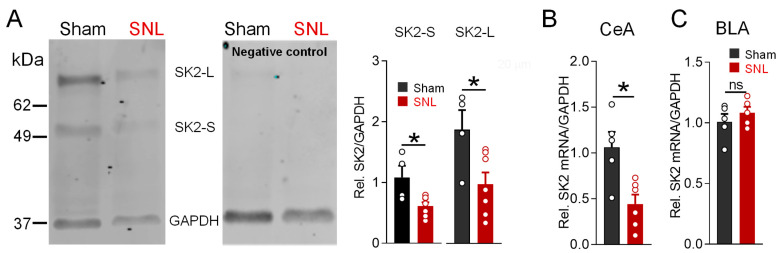
SK2 protein and mRNA expression in the CeA is decreased in chronic neuropathic pain. (**A**) Western blotting analysis of SK2 channel short (SK2-S) and long (SK2-L) isoforms in CeA lysates from sham and neuropathic rats 4 weeks post spinal nerve ligation (SNL) surgery. Blots were re-probed after incubation with the SK2 subunit-blocking peptide as a negative control. The levels of both SK2 protein isoforms were significantly decreased in neuropathic as compared to the sham control rats (* *p* < 0.05, unpaired *t*-test; *n* = 4 sham, *n* = 7 SNL rats). (**B**,**C**) RT-PCR analysis of SK2 mRNA expression level in CeA (**B**) and BLA (**C**). CeA SK2 mRNA level decreased in SNL rats compared to the sham controls (* *p* < 0.05, unpaired *t*-test, *n* = 5 sham rats, *n* = 6 SNL rats). In BLA, SK2 mRNA expression level remained unchanged (ns, *p* > 0.05, unpaired *t*-test, *n* = 5 sham rats, *n* = 5 SNL rats).

To determine if the impaired SK channel function observed in the behavioral and electrophysiological experiments was due to decreased channel expression, the protein levels of the predominant SK channel subunit (SK2) in the amygdala [17] were determined via Western blotting with a whole-cell lysate preparation of the right CeA tissue. The right CeA was selected because of evidence of the right-hemispheric lateralization of neuropathic pain processing [8]. The α-SK2 antibody recognized two isoforms of SK2 protein in the amygdala (CeA): short (SK2-S) and long (SK2-L), as previously reported in mouse and human brains [76,77]. The protein levels of the SK2-S and SK2-L isoforms decreased by more than 50% in the CeA of SNL rats compared to the sham control rats (Figure 5A, bar histograms, *p* < 0.05, unpaired *t*-tests, sham *n* = four rats, SNL, *n* = seven rats).

To determine if the decrease in SK2 protein levels was due to a transcriptional mechanism, we measured SK2 mRNA levels using reverse transcription polymerase chain reaction (RT-PCR). Right CeA samples isolated from SNL rats (*n* = 6 samples) showed decreased (42% of sham values) SK2 subunit mRNA levels compared to the sham controls (*n* = five samples, Figure 5B, *p* < 0.05, unpaired *t*-test), suggesting the downregulation of SK2 expression through a pre-transcriptional mechanism. To confirm the regional selectivity of transcriptional SK2 dysregulation in the CeA, we measured SK2 mRNA levels in the neighboring BLA. Right BLA tissue samples isolated from SNL rats showed no difference in SK2 mRNA levels compared to the sham controls (Figure 5C, *p* > 0.05, unpaired *t*-test, *n* = 5 samples per group), suggesting that the pre-transcriptional SK channel mRNA downregulation in the amygdala was localized specifically to the CeA.

**Figure 6:**
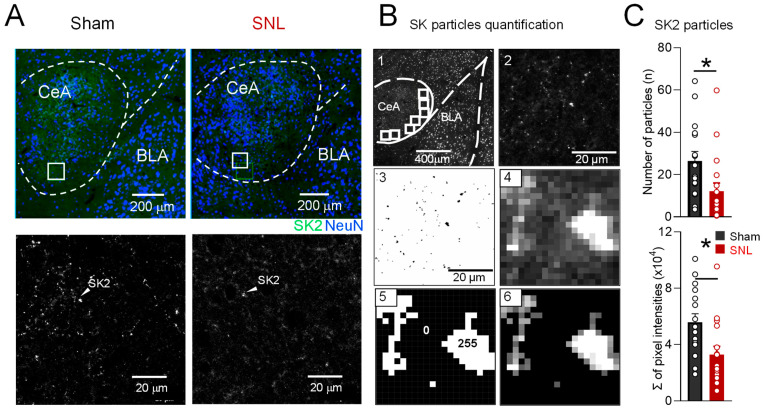
Expression of SK2 protein is decreased in lateral capsular central amygdala (CeLC) in chronic neuropathic pain. (**A**) Representative images of coronal CeA sections immunostained for SK2 (green) and neuronal nuclei (NeuN, blue) protein. White squares in the top images depict the CeLC area shown in larger magnification on the bottom. Arrowheads indicate individual SK2 particles. (**B**) Analysis protocol for SK2 protein quantification in the CeLC. The amygdala was identified by counterstaining neurons with NeuN protein antibody. The CeLC division was filled with square sample (B1) areas to be imaged at a high resolution (B2). High-resolution image was segmented via thresholding (B3) to calculate number and size of SK2-positive particles. For raw integrated density analysis, a raw SK2 image (B4) was segmented with a binary mask (B5) to assign 0 (black) to pixels with a non-specific signal in the resulting image (B6). (**C**) Number of labeled SK2 particles (top graph) and total intensity of SK2 protein (bottom graph) in the CeLC were decreased in neuropathic (SNL) as compared to sham rats. (* *p* < 0.05, unpaired *t* test; *n* = 5 sham, *n* = 5 SNL rats). Abbreviations: CeA, central amygdala; BLA, basolateral amygdala.

Since the brain slice physiology experiments focused on CeLC neurons, we analyzed SK2 protein expression levels in this region using immunohistochemistry. Sections containing CeA were immunostained for SK2 (green) and neuronal nuclei (NeuN, blue) protein (Figure 6A top). Representative high-resolution black and white images show numerous SK2 particles labeled in a section from a sham rat but only scarce particles in a similar region in a section from an SNL rat (Figure 6A bottom). Using ImageJ software (ImageJ v1.53f51, NIH), we calculated the number and size of SK2 particles, as well as the total intensity of the signal for SK2 channels in the CeA of SNL and sham rats (Figure 6B). CeLC was filled with sample areas (Figure 6B-1) and imaged at a high resolution (Figure 6B-2) to calculate the number and size of SK2 particles per sample area (Figure 6B-3). For total intensity analysis, a raw SK2 image (Figure 6B-4) was segmented with a binary mask (Figure 6B-5) to assign 0 (black) to pixels with a non-specific signal in the resulting image (Figure 6B-6). We found that the total number of SK2 particles in the CeA of SNL rats was lower than in the sham controls (Figure 6C top graph, *p* < 0.05, unpaired *t*-test, *n* = 5 rats in each group) and that the sum of pixel intensities was significantly lower in the CeA of SNL rats compared to the sham rats (Figure 6C bottom graph, *p* < 0.05, *n* = 5 rats in each group, unpaired *t*-test), consistent with the decreased number of SK2 particles in the CeA of SNL rats. However, we found no change in average SK2-positive particle size in the CeA of sham and SNL animals (0.102 ± 0.004 µm, 0.094 ± 0.005 µm, respectively; *p* > 0.05, unpaired *t*-test).

### 3.5. DNA Methylation Profile of CpG Island in the SK2 Gene Promoter Region in CeA

**Figure 7:**
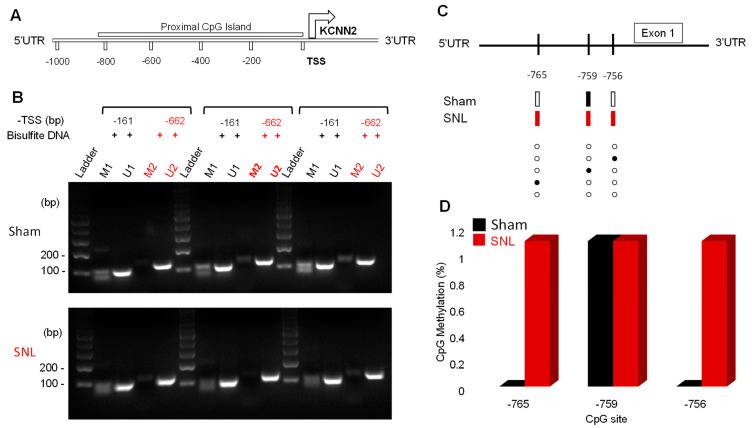
Methylation pattern of CpG island of SK2 promoter region at target sites in CeA tissue. (**A**) Schematic of the SK2 gene (KCNN2) promoter region and proximal CpG island 850 bp upstream of the transcription start site (TSS). (**B**) Methylation-specific PCR using primers designated as methylated (M1 and 2) and unmethylated (U1 and 2) spanning positions −161 bp and −662 bp upstream of the TSS of SK2 gene in bisulfite-converted DNA samples obtained from sham and SNL rat CeA tissue samples. DNA amplicon sizes were determined using a 100 bp DNA ladder. (**C**) CpG methylation status of SK2 promoter region spanning −765 to −756 upstream of the TSS from different clones in the samples from sham and SNL rats. Methylated sites are depicted in closed rectangles/circles, and unmethylated site in open rectangles/circles. Only 5/10 clones are displayed; 5 clones with non-methylated sites are not shown for better clarity. (**D**) CpG methylation percentage at positions −765 to −756.

To better understand the pre-transcriptional mechanism of SK channel downregulation, we examined the relationship between SK2 gene promoter methylation and reduced protein expression by determining the CpG methylation pattern of the approximately 850 bp region within the promoter of SK2 gene (KCNN2) in right CeA tissue samples from sham (*n* = 9) and SNL (*n* = 9) rats (Figure 7A). Ten clones were analyzed per CeA sample for the methylation of CpG dinucleotides within the promoter region proximal to the transcription start site (TSS).

Methylation-specific PCRs were carried out to determine methylated regions of the CpG islands within the promoter region of the SK2 gene. We found that the promoter regions analyzed were largely unmethylated (Figure 7B). The amplification of expected amplicon size of ~102 (M1/U1) and 120–124 bp (M2/U2) occurred in all the samples analyzed and demonstrated that methylated or unmethylated DNA could be recognized and distinguished by using specific primer sequences. Bisulfite sequencing analysis revealed a generally low methylation level across the CpG island between the SNL and sham rat samples; however, we noticed a difference in the methylation sites between the two groups. Interestingly, a sequence of three CpG dinucleotides was methylated in SNL rats at positions −765, −759, and −756 relative to the TSS (Figure 7C). Of the three sites, only the CpG at −759 was methylated in the sham rat CeA. We measured the percentage methylation of these CpG dinucleotides across all the clones and observed a methylation frequency of ~1.1% (Figure 7D).

**Figure 8:**
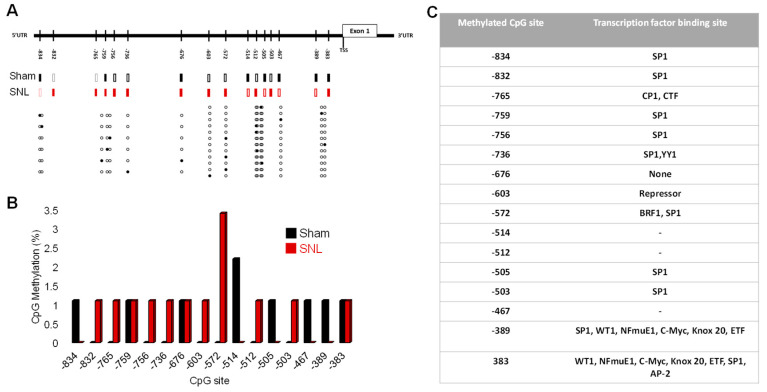
Increased differential methylation of CpG islands of SK2 promoter region in the CeA in neuropathic pain. (**A**) CpG methylation status of SK2 promoter region spanning −834 to −383 upstream of the transcription start site from different clones in the samples obtained from SNL rats. Methylated sites are depicted in closed rectangles/circles and unmethylated site in open rectangles/circles. (**B**) CpG methylation percentage at positions −832 to −383 in samples from sham and SNL rats. (**C**) Table showing transcription factor binding sites at each methylated CpG in samples from sham and SNL rats.

We extended the analysis to other methylated CpG sites in the CeA of SNL rats and observed a total of 11 CpG sites ranging from position −834 to −383 relative to the TSS of SK2 gene (Figure 8A). Of the eleven sites identified, three sites were also found to be methylated in the CeA of sham rats (−759, −676, and −383) (Figure 8A). We observed methylation levels of ~3.3% at position −572 in the SNL relative to the sham rats, whereas a level of 1.1% methylation was observed across the rest of the CpG sites identified when compared to the sham rats (Figure 8B). We found that −834, −514, −505, −467, and −389 CpG sites were specifically methylated in the CeA of sham rats but not in SNL rats. Methylation levels ranged from 2.2% at position −514 to 1.1% at other CpG sites in sham relative to SNL rats (Figure 8B). The total methylation level was elevated in the CeA of SNL rats (14.3%) as compared to the sham rats (9.9%).

Analysis of transcription factor consensus sequence binding sites associated with these dinucleotides was performed with the Alibaba2 gene regulation program. We identified different transcription factor binding sites across SNL-specific methylated CpGs (Figure 8C). Interestingly, we found many transcription factors from different families. These include the CCAAT binding family of proteins (CP1 and CTF), the ubiquitous specificity protein factor (Sp1), which belongs to the Sp/Krupper-like factor family of transcription factors, Yin Yang-1 (YY1) of the GL1-Kruppel class of zinc finger proteins, TFIIB-Related Factor (BRF1), Wilms tumor suppressor/transcriptional repressors (WT1), mouse B-cell nuclear factor (NF-muE1), Myc family of proteins (C-Myc), early growth response protein 2 (Knox-20), transcription enhancer factor (TEA), domain-containing factor (ETF), repressor protein, as well as activating protein-2 family of transcription factors (AP-2). A common transcription factor binding site across sham-specific methylated CpG sites was the Sp1 factor (Figure 8C). At position −389, more transcription factor binding sites for WT1, NF-muE1, C-Myc, Knox-20, and ETF transcription factors were predicted.

The data suggest that increased DNA methylation and different methylated CpG sites of CPG island of the SK2 promoter region in the CeA in neuropathic pain contribute to the epigenetic silencing of the SK2 gene (KCNN2).

## 4. Discussion

This study advances the novel concept that amygdala neurons undergo maladaptive plasticity in chronic neuropathic pain due to the loss of SK channel function. Altered electrophysiological profiles of SK channel function in CeLC neurons were associated with decreased levels of SK2 protein due to altered SK2 gene (KCNN2) expression through a pretranscriptional mechanism that involves epigenetic dysregulation. Suppressed SK2 mRNA expression resulting in decreased SK protein expression produced abnormally enhanced neuronal function and pain-like behaviors through the loss of inhibitory control. These findings are significant because they identify SK2 channel dysfunction as a mechanism of hyperexcitability in chronic pain involving epigenetic changes.

This study advances our understanding of brain mechanisms of chronic pain in several ways. Neuronal excitability in the CeA has been reported to be potentiated in various pain models but was shown mostly at the relatively acute stages in acute arthritis [65,78], inflammatory [79,80], visceral [81] and neuropathic pain [10,29,44] models. The present data provide important mechanistic insights into chronic neuroplasticity by identifying impaired SK channel function in CeLC neurons in chronic neuropathic pain as a major factor. We found a pain-related decrease in SK2 channel-mediated mAHP/I_AHP_, resulting in an increase in the excitability of a certain type of CeLC neurons (regular-spiking). Changes in neuronal membrane properties due to a decrease in the expression of SK2 channels were recently demonstrated in the hypothalamic paraventricular nucleus in a model of repeated neonatal visceral pain [46]. Our results show that the loss of SK channels in a different key component of the limbic system, the CeA, induces hyperexcitability to promote pain behaviors. Amygdala–hypothalamic interactions are well established [82], but further studies are necessary to understand their role in chronic pain. Chronic stress conditions have been shown to cause decreased mAHP and hyperexcitability in pyramidal BLA neurons [83]. Since we did not find changes in SK channel expression in the BLA (Figure 5C), our findings may be pain-specific or different mechanisms may drive hyperexcitability in BLA and CeA.

CeA neurons in the lateral and capsular divisions are unique because they receive monosynaptic nociceptive input from the PB [3,11,84,85,86]. CRF-, PKCδ-, and some somatostatin-positive neurons in the CeA receive direct projections from the PB [27,87], and all three cell types project to various brain regions important for pain modulation [1,33,88]. Our recordings were limited to unidentified regular-spiking neurons in the CeLC, which were the first to be shown to develop synaptic plasticity and hyperexcitability in an acute arthritis pain model by our group [88]. We targeted the CeLC, where PKCδ-positive neurons represent the major population [29,89,90], and the increased excitability of PKCδ neurons at the acute stage of a neuropathic pain model has been linked to the amplification of pain-related behaviors in rodents [29]. Somatostatin-positive neurons in the CeLC were also found to play a critical role in fear, defensive behavior, and pain modulation [91,92], but their excitability is decreased in neuropathic pain, shifting the excitatory balance toward PKCδ neurons [27,29]. These findings suggest that SNL-induced loss of SK channel function may be specific to PKCδ neurons. Future studies examining cell-specific differences in this region in the context of SK channel function and plasticity in pain may yield important information about the regulation and roles of these neurons. As an example, we found that adapting burst-firing cells, another type found in the lateral CeA [24,30], do not undergo SK channel dysfunction under neuropathic pain. These neurons exhibit a prominent slow AHP [24], whose function in pain processing has yet to be determined.

Three types of SK channels may contribute to neuronal excitability in the CeA and can regulate amygdala-dependent behaviors. Lateral CeA neurons predominantly express SK2 and SK3 subtypes [17,18], and SK2 is the most apamin-sensitive. The results from our immunohistochemical and immunoblotting experiments suggest the specific downregulation of the SK2 protein in the CeA. Interestingly, the expression of both SK2 protein isoforms (SK2-S and SK2-L) is decreased in the CeA (Figure 5A). Although the distinct roles of these two SK2 isoforms in the CeA in pain processing have yet to be established, some evidence suggests differential functions. SK2-S predominantly mediates the apamin-sensitive mAHPs following somatic depolarization, whereas SK2-L is predominantly localized in dendritic spines [93], and NMDA-dependent inhibitory function of dendritic SK channels on evoked synaptic activity in the hippocampus has been reported [19].

Our study further demonstrates that the increase in the SK channel-mediated mAHP with NS309 in regular-spiking CeLC neurons decreases hyperexcitability and alleviates pain-induced averse-affective behaviors (vocalizations) but not peripheral mechanosensitivity (von Frey test). The reason for this differential effect remains to be determined but may point to spinal sensory mechanisms and extra-amygdala projections. SNL produces lasting tactile allodynia measured as spinal reflex responses that can be modulated by higher-mediated brain centers, such as the amygdala. [94,95,96,97]. Higher integrated averse-affective behaviors, such as those assessed by measuring vocalizations, are generated in brain circuits involving the amygdala. Audible vocalizations evoked due to noxious stimuli were found to reflect higher-organized nociceptive responses [98,99]. Low-frequency ultrasonic vocalizations (~25 kHz) indicate negative affective responses [51,52,100]. In a chronic pain model, noxious stimulus-evoked vocalizations represent emotional responses to a noxious stimulus in a pain condition and serve as a measure for the emotional component of pain that characterizes pain according to the official definition by the International Association of the Study of Pain [101]. It is well documented that the amygdala integrates nociceptive signals with emotional processing [1,10,102]. In this study, audible and ultrasonic vocalizations were decreased by reducing amygdalar hyperexcitability, indicating an important role of SK channels in the amygdala in the control of averse-affective behaviors rather than the modulation of mechanosensitivity. Some manipulations of CeA activity have been shown to modulate both mechanosensitivity and averse-affective behaviors, whereas others, such as opioids, largely do not, or not consistently, affect mechanosensitivity [88], and pharmacological or optogenetic activation of CeA neurons can decrease the responses of spinal neurons to innocuous stimuli, implicating the amygdala in the descending modulation of sensory aspects of pain [103].

Our real-time RT-PCR experiments revealed that the SK2 channel transcript was strongly downregulated. This downregulation was localized to the CeA, and no change was observed in the neighboring BLA. Unlike CeA, the BLA does not receive direct nociceptive input through the PB but has been implicated in the processing of nociceptive information from the limbic cortex, thalamus, and periaqueductal gray matter [104,105,106,107]. An interesting explanation for subregion-specific SK channel dysregulation in the amygdala is that of increased or altered PB-CeLC synaptic transmission. The contribution of changes in BLA input to SK channel dysfunction in the CeA remains to be determined.

The epigenetic regulation of gene expression that brings about experience-dependent plasticity is reversible and dynamic [108,109,110]. Promoter DNA methylation regulates the expression of a gene either by impeding the interactions of transcription factors with DNA or by recruiting epigenetic readers that bind to methylated DNA [111]. Epigenetic silencing is a natural phenomenon, in which gene expression is regulated through DNA modifications. DNA methylation is a common mechanism that cells use to silence the transcription of genes [112]. DNA methylation has been linked to chronic pain and cognitive impairment [113,114,115,116]. A major knowledge gap is how nerve injury induces and maintains chronic pain. Here, we suggested a role of methylation in the transcriptional silencing of inhibitory potassium channels (SK2) associated with neuropathic pain in the CeA. We assessed the methylation profile of the SK2 gene promoter region in a neuropathic pain model compared to sham rats using bisulfite sequencing methods. Several interesting observations were made. While methylation levels were generally low, methylated CpG sites identified in the CeA samples from SNL rats were different from those detected in sham rats. While there is no consensus on the cut-off point for determining hypermethylation or hypomethylation, in the context of this study, we determined the DNA methylation level by averaging the methylation values for each CpG site. The low methylation level across the CpG Island is in reference to the total methylation level of 14.3% in the case of SNL and 9.9% in sham CeA rats. Although the function of each methylated CpG site remains to be determined, using in silico methods, we showed that a number of transcription factor consensus binding sites exist around these methylated CpGs (Figure 8). The majority of these transcription factors have dual functions and can either activate or repress the expression of a gene depending on the promoter context in which they are found. These include the CP1, CTF, Sp1, YY1, WT1, and C-Myc transcription factors. On the other hand, we identified repressor proteins that prevent gene transcription by binding to the promoter region. We also found other transcription factors involved in gene activation, namely BRF1, NF-muE1, Knox-20, ETF/Tead2 and AP-2 [117,118,119]. Several of these transcription factors are expressed in brain tissues under different scenarios linked with cellular responses to diverse physiologic stresses [120,121,122,123]. Further studies are needed to determine the expression of these transcription factors, the effect of methylation on the DNA binding ability of these transcription factors, and their role in modulating the expression or repression of the SK2 gene in the amygdala under normal conditions and in neuropathic pain.

DNA demethylation is a promising yet challenging therapeutic target for the treatment of numerous disorders, including pain [116,124]. DNA methylation is mediated by methyltransferases. Commercially available inhibitors of DNA methyltransferases have been recently studied in neuropathic pain conditions. RG108, a non-nucleoside inhibitor, decreased methylation and blocked mechanical allodynia by restoring the expression of the K2P1.1 channel in the dorsal root ganglia [125]. However, another inhibitor (RG107) induced pain behaviors by activating the transcription factor (CREB) to methylate the KV1.2 promoter region [126]. Potent nucleoside inhibitors (5-azacytidine and zebularine) prevented the methylation of the miR-214-3p promoter, alleviating neuropathic and neonatal pain behaviors [127,128]. The therapeutic implications and usefulness of DNA methylation and demethylation remain to be determined.

As a caveat, this study used only males. The rationale was that our hypothesis of SK channel dysfunction as a contributor to amygdala hyperexcitability and neuropathic pain behaviors was built on studies that showed pain-related neuroplasticity in the amygdala, and these studies were carried out almost exclusively using males [1,47]. This does not negate the importance of studying both males and females; however, sex differences were not the goal of this study, and future studies are necessary to understand if similar changes and mechanisms of SK dysregulation are observed in females. This study provides the scientific premise for these important but more complex experiments.

## 5. Conclusions

This study shows that chronic neuropathic pain behaviors involve the increased excitability of CeA neurons due to the loss of SK2 function. Increased DNA methylation of the CpG island of the SK2 promoter region and different methylated CpG sites in chronic neuropathic pain contribute to the epigenetic silencing of the SK2 gene, decreased SK2 mRNA transcripts and protein expression, the dysfunction of inhibitory control and maladaptive hyperexcitability in the CeA, and neuropathic pain behaviors. Restoring inhibitory SK signaling in the amygdala could open new ways to mitigating chronic neuropathic pain.

## Figures and Tables

**Table 1:** Primers used for bisulfite-sequencing-specific PCR and cloning.

**Methylation-Specific Primers**	**Sequence**	**Amplicon Size**
M1-left	GGGTAGTTAGTTTAATGTGAGCGA	102 bp
M1-right	TAATAATACAAAAAAACGAACGCG	
U1-left	GGGGTAGTTAGTTTAATGTGAGTGA	102 bp
U1-right	AATAATACAAAAAAACAAACACAAA	
M2-left	GTGCGTTTAATTAATCGGATTC	120 bp
M2-right	GATAATACACCCTACGCAATACGTT	
U2-left	TTGGTGTGTTTAATTAATTGGATTT	124 bp
U2-right	CAATAATACACCCTACACAATACATT	
**Bisulfite-Specific Primers**		
BSP-F1	AGGGGTTTTTATTTTGTAGG	285 bp
BSP-R1	ACAATAAAATTTCCATATCAAACCTAT	
BSP-F2	AATAGGTTTGATATGGAAATTTTA	287 bp
BSP-R2	AACAACAAAAACAAATTATCCCC	

## Data Availability

The datasets generated and/or analyzed during the current study are available from the corresponding author upon reasonable request.
